# Multi-compartment scaffold fabricated via 3D-printing as *in vitro* co-culture osteogenic model

**DOI:** 10.1038/s41598-018-33472-1

**Published:** 2018-10-11

**Authors:** Elvira De Giglio, Maria A. Bonifacio, Ana M. Ferreira, Stefania Cometa, Zhi Yuan Ti, Antonella Stanzione, Kenny Dalgarno, Piergiorgio Gentile

**Affiliations:** 1 Department of Chemistry, University of Bari Aldo Moro, Via E. Orabona 4, Bari, 70126 Italy; 2 School of Engineering, Newcastle University, Stephenson Building, Claremont Road, Newcastle upon Tyne, NE1 7RU UK; 3 Jaber Innovation s.r.l., via Calcutta 8, Rome, 00144 Italy

## Abstract

The development of *in vitro* 3D models to get insights into the mechanisms of bone regeneration could accelerate the translation of experimental findings to the clinic, reducing costs and duration of experiments. This work explores the design and manufacturing of multi-compartments structures in poly(ε-caprolactone) (PCL) 3D-printed by Fused Filament Fabrication technique. The construct was designed with interconnected stalls to host stem cells and endothelial cells. Cells were encapsulated within an optimised gellan gum (GG)-based hydrogel matrix, crosslinked using strontium (Sr^2+^) ions to exploit its bioactivity and finally, assembled within compartments with different sizes. Calcium (Ca^2+^)-crosslinked gels were also used as control for comparison of Sr^2+^ osteogenic effect. The results obtained demonstrated that Sr^2+^ ions were successfully diffused within the hydrogel matrix and increased the hydrogel matrix strength properties under compressive load. The *in vitro* co-culture of human-TERT mesenchymal stem cells (TERT- hMSCs) and human umbilical vein endothelial cells (HUVECs), encapsulated within Sr^2+^ ions containing GG-hydrogels and inter-connected by compartmentalised scaffolds under osteogenic conditions, enhanced cell viability and supported osteogenesis, with a significant increase of alkaline phosphatase activity, osteopontin and osteocalcin respect with the Ca^2+^-crosslinked GG-PCL scaffolds. These outcomes demonstrate that the design and manufacturing of compartmentalised co-culture of TERT-hMSCs and HUVEC populations enables an effective system to study and promote osteogenesis.

## Introduction

The repair of bone defects occurs through a complex cascade of events which could be studied by *in vivo* experiments on animal models^1,2^. However, many ethical concerns arise from the use of animals, in addition to high costs. Furthermore, the complexity and duration of such experiments has encouraged the development of suitable *in vitro* bone models, useful to simulate the native three-dimensional (3D) environment better than traditional two-dimensional (2D) cell cultures. Indeed, the architecture of a 3D system has a significant impact on cell behaviour, as well as its geometry^3^. In this respect, a previous study reported that bone tissue regeneration was enhanced by surface curvature, particularly by concave structures^4^. Moreover, Bidan *et al*. studied the impact of pores geometry on bone growth, providing a theoretical model to explain the improvement of tissue deposition in circular pores rather than other shapes^5^.

The development of complex 3D networks for *in vitro* studies is a stimulating challenge which involves the design and fabrication of intricate structures. To provide custom-made 3D geometries, a promising approach is the use of additive manufacturing techniques that lead to complex structures, starting from an STL file format. 3D printing techniques possess several advantages with respect to the conventional fabrication approaches (e.g. freeze-drying, solvent casting, porogen leaching, gas foaming), that are mainly affected by limitations in shape complexity. Recently, moulding and machining methods were proposed for the obtainment of improved final structures through material removal processes. However, the latter are too expensive and difficult to control^6^. In contrast, additive manufacturing techniques are flexible and cost-effective, allowing the complete customization of the printed scaffold structure^7^. Different 3D printing techniques can be used in the biomedical field to create tailored implants, devices, *in vitro* models and drug-release systems^8^. As an example, selective laser sintering is a technique which allows to easily print complex geometries starting from the powder of a polymer or metal, without support materials^9,10^. However, this techniques often require post-processing to smooth the resulting grainy surfaces. In addition, several alternative techniques allow to print at a faster rate^11^. Therefore, among the wide range of additive manufacturing approaches, Fused Filament Fabrication (FFF), consisting in the layer-by-layer extrusion of a thermoplastic filament, is the most employed technology to fabricate medical implants and provides several advantages: (1) low cost, combined with the high speed of scaffold fabrication, simple material processing and ease of usage, which makes reasonable the scale-up and the translation to the “bedside manufacturing”; (2) reduced materials residence time in the heating step; (3) development of scaffolds with 100% pores interconnectivity, fundamental for cell growing and diffusion of nutrients; (4) tuneable printing quality through the accurate optimization of working parameters (e.g. infill ratio and geometry, nozzle and build-plate temperature, printing speed and layer thickness)^12,13^.

The aim of this study was to fabricate a polymeric 3D printed scaffold as *in vitro* model with a new multi-compartment design for co-culture of two different cell populations. *In vitro* co-culture systems allow to explore cell-cell interactions, overcoming the limitations of traditional monoculture experiments^14^ and becoming useful tools as drug discovery platforms, as well as for tissue engineering and regenerative medicine^15,16^. The role of the extracellular microenvironment on cell interactions could be investigated with a co-culture setup, exploiting several approaches ranging from spheroids preparation to microfluidics techniques^15^. In this respect, some interesting results were reported by Bersini and coworkers, who studied the impact of several parameters on human umbilical vein endothelial cells (HUVECs) and MSCs in an *in vitro* 3D co-culture model^17^. Other authors described the preparation of spheroids systems where they showed the positive effect of HUVECs on MSCs osteogenesis^18,19^. However, at the best of our knowledge, a 3D-printed system for the co-culture of HUVECs and hMSCs could not be found in literature.

**Figure 1:**
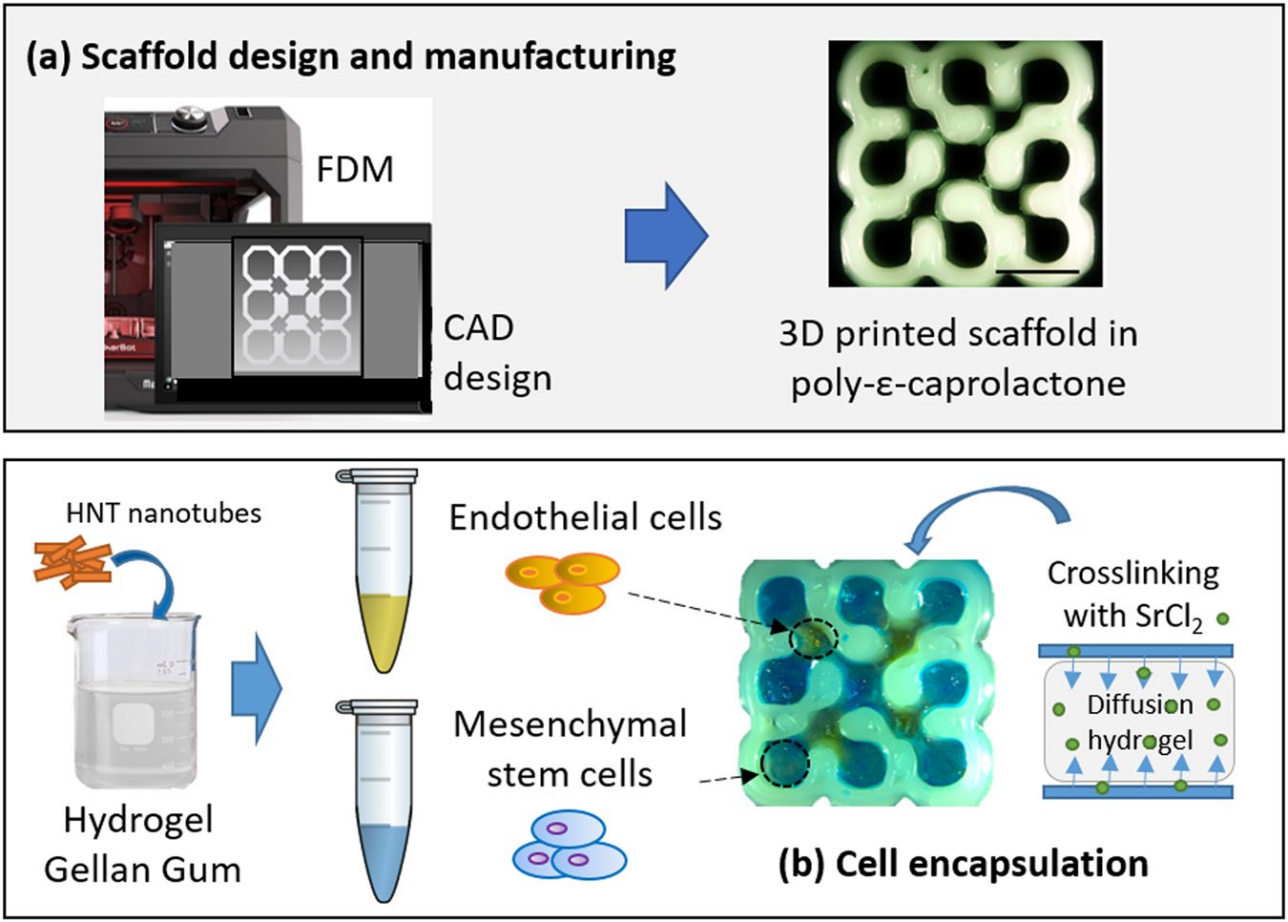
Schematic representation of the 3D construct preparation. (**a**) From CAD design by Autodesk Inventor Professional software to the 3D printed PCL scaffold by MakerBot Replicator 2X. (**b**) Cell encapsulation in gellan gum-based hydrogels.

Herein, we designed an *in vitro* co-culture model (Fig. 1) with interconnected compartments as pores with different sizes to host two cell types, stromal mesenchymal stem cells and endothelial cells. Hence, a 3D-printed PCL structure was designed with a two different sized compartments, relatively easy to print and scalable as a simple periodic unit.

To print designed 3D PCL scaffolds, the MakerBot replicator 2X printer was selected since it has been already used by physicians to fabricate 3D models in clinical practice. Recently, Albrecht *et al*. exploited a MakerBot printer to fabricate 3D scaffolds for bone tissue engineering, extruding PCL filaments in a log-pile geometry^20^. Indeed, PCL is often used as thermoplastic polymer to fabricate bone substitutes, because of its ease of processability, suitable mechanical properties and slow degradation profile^21^. However, like many synthetic polymers, it is characterized by poor cellular adhesion^22–24^.

In this study, we decided to assemble a gel matrix within a 3D-printed PCL structure to provide a more suitable microenvironment for cells, enhancing the biocompatibility of the construct through ECM mimic. In a previous work, a gellan gum (GG)-based hydrogel has been developed by these authors for tissue engineering applications, optimizing its biocompatibility and stiffness^25,26^. The GG matrix was associated with an inorganic filler (halloysite nanotubes, HNT) and showed effective mechanical and biocompatible features^27^.

Furthermore, herein we exploited the GG hydrogel to encapsulate stromal mesenchymal stem, for bone tissue formation, and the influence of the co-culture of endothelial cells, into a 3D-printed scaffold. In literature, only few attempts of cell encapsulation within hydrogels poured in traditional log-pile 3D scaffolds can be found^20,28^. The complex construct reported in this work represents, at the best of our knowledge, the first assembly of a cell-embedding hydrogel in a complex, 3D printed backbone, carefully designed to help studying *in vitro* cells behaviour.

Furthermore, cell-embedded GG hydrogels were recently proposed as innovative, injectable systems for nucleus pulposus regeneration, cartilage repair and therapeutic cell and drug delivery^29–31^. Beyond the state of the art, the already developed hydrogels were crosslinked with Sr^2+^ ions to evaluate its impact on mechanical and biological features. The use of Sr^2+^ ions as crosslinkers for hydrogels has been exploited due to its demonstrated bioactivity and stimulating effect on bone formation and angiogenesis, *in vitro* and *in vivo*^32–36^. A traditional ionic crosslinking approach with Ca^2+^ ions has been also adopted as control.

This proposed compartmentalised 3D scaffold model has potentiality for studying cell behaviour in co-culture systems for understanding bone diseases, drug testing and formations of vascularised micro-tissues. The fabricated construct was characterised in terms of structure and physico-chemical properties. Furthermore, mechanical tests analysis (static compression and stress-relaxation analyses) were performed in combination with high-order finite element methods for studying stress-strain distribution for further tissue engineering applications. Human TERT immortalised bone marrow stromal cell line (Y201) and HUVEC were encapsulated into the GG hydrogel and allocated into interconnected compartments within a 3D-printed scaffold to evaluate the biological properties and osteogenesis response of the co-culture in the composite construct.

## Results and Discussion

### FFF process parameter optimisation and morphological analysis of the PCL scaffolds and GG-PCL constructs

**Figure 2:**
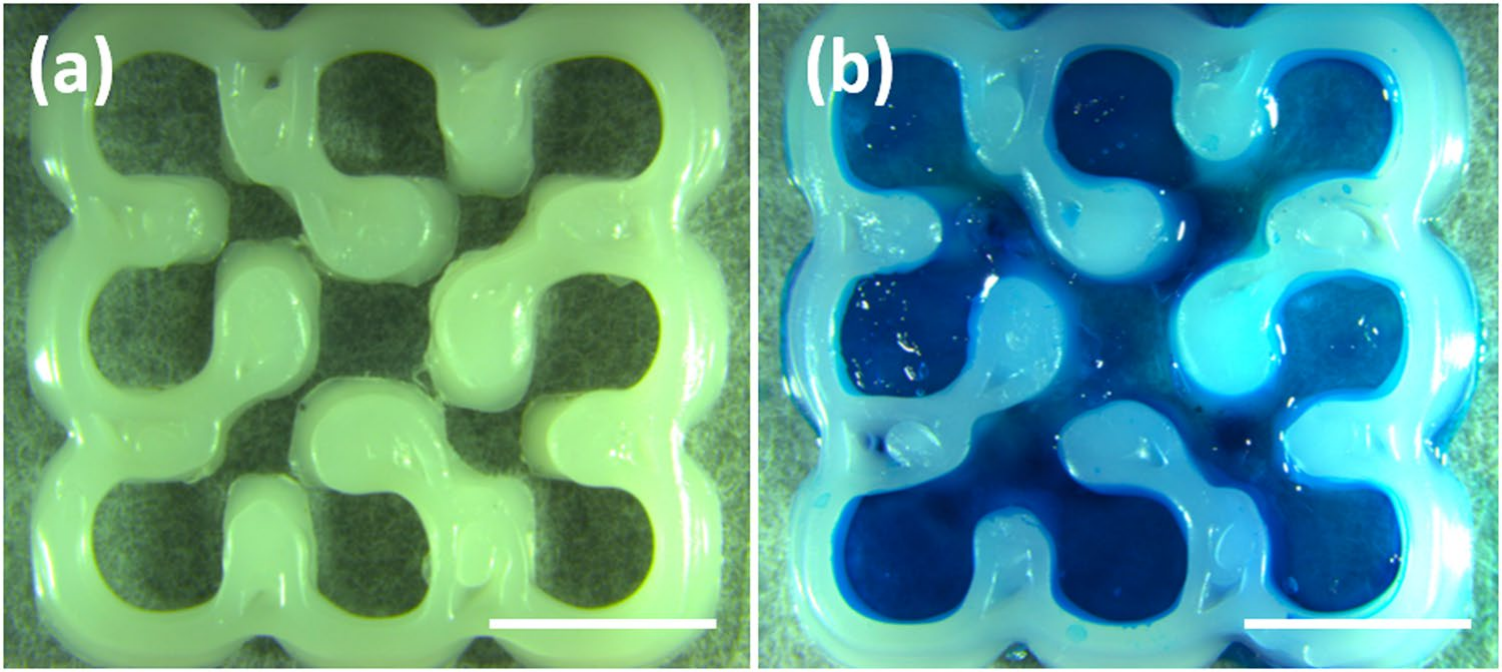
Stereomicroscope images of (**a**) 3D PCL printed scaffold; (**b**) 3D GG-PCL construct. The hydrogel was stained with a blue food colour for improving the visualisation at the stereomicroscope. Scale bars correspond to 5 mm.

Stereomicroscope images of the 3D network scaffold printed by MakerBot replicator 2X are shown in Fig. 2. The construct was printed after optimising the PCL filament extrusion (achievement of consistent 1.75 mm diameter that is a necessary requirement for several commercially available 3D printers) and the printing conditions (speed was varied from 25 to 250 mm∙min^−1^, extruder temperature in range from 120 to 140 °C and suckback from 0 to 5000 µm at 25 mm∙min^−1^). This was in accordance with the MakerBot optimisation process described also by Albrecht *et al*.^20^, where the authors 3D printed PLA and PCL porous scaffolds at a velocity of 25 mm/s setting the suckback as zero in order to avoid the printer nozzle block due to the high PCL specific heat. The obtained PCL scaffold showed the designed combination of pores with a size of 2.0 ± 0.2 mm) and 0.8 ± 0.1 mm for the encapsulation of stromal mesenchymal stem and endothelial cells respectively. The design of these circular-shape pores were in accordance with Bidan *et al*. reported in their work where an increase of bone growth has been described rather than semi-circular shapes^5^.

### X-ray photoelectron spectroscopy (XPS analysis)

XPS analysis of both the GG- and PCL-based materials revealed the elemental composition reported in Table 1. As far as the PCL is concerned, the elemental composition is typical of the polymer^37^, except for the negligible amount of silicon detected, likely due to manufacturing processes. Furthermore, XPS analysis of the GG showed the main contributions of carbon and oxygen, ascribable to the polymeric matrix. Moreover, the aluminium and silicon signals, characteristic of halloysite nanotubes, were found in the expected 1:1 stoichiometry. The presence of strontium was provided by the crosslinking procedure.

**Table 1:** Atomic percentages (At%) of GG- and PCL-based moieties of GG-PCL constructs by XPS analysis.

Material	Element (At%)				
	C_1s_	O_1s_	Al_2p_	Si_2p_	Sr_3d_
**PCL**	74.1 ± 0.5	21.2 ± 0.6	—	4.7 ± 1.2	—
**GG**	50.3 ± 0.2	48.4 ± 0.3	0.4 ± 0.1	0.5 ± 0.2	0.4 ± 0.1

### Water uptake evaluation

**Figure 3:**
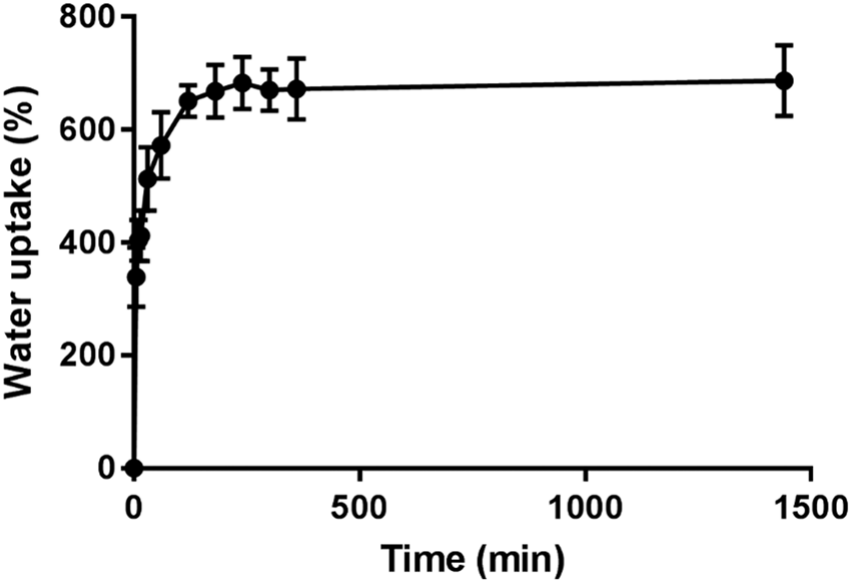
Water uptake of GG-PCL samples (n = 3).

The swelling of the GG-PCL constructs was studied in PBS at 37 °C and the water uptake profile over 24 h was reported in Fig. 3. The study of PCL and GG moieties alone revealed that only the gellan-based ones experienced the swelling process, while the PCL behaviour was negligible, as expected. The GG crosslinked with Sr^2+^ ions resulted completely swelled after 2 h, while the same gel crosslinked with Ca^2+^ rapidly swelled upon contact with PBS, reaching a maximum just after five minutes as reported in an our previous work^25^. Moreover, the water uptake at 24 h experienced by the Sr^2+^-crosslinked gel resulted lower than that observed for the Ca^2+^-crosslinked gel (i.e., 690 vs 840%). The latter results, associated with those of compression tests, indicated that stiffer gels could be achieved by Sr^2+^ ions crosslinking.

### Mechanical properties of GG hydrogels, PCL scaffolds and GG-PCL constructs

Static compression tests were performed in order to measure the mechanical properties of all the constructs for evaluating the influence of GG hydrogels on the PCL structure. Hydrogels are attractive matrices to enhance cell-based therapies for tissue repair due to their injectability and ease of filling defects of any shape, as well as to modify the mechanical properties of composites^38^.

**Figure 4:**
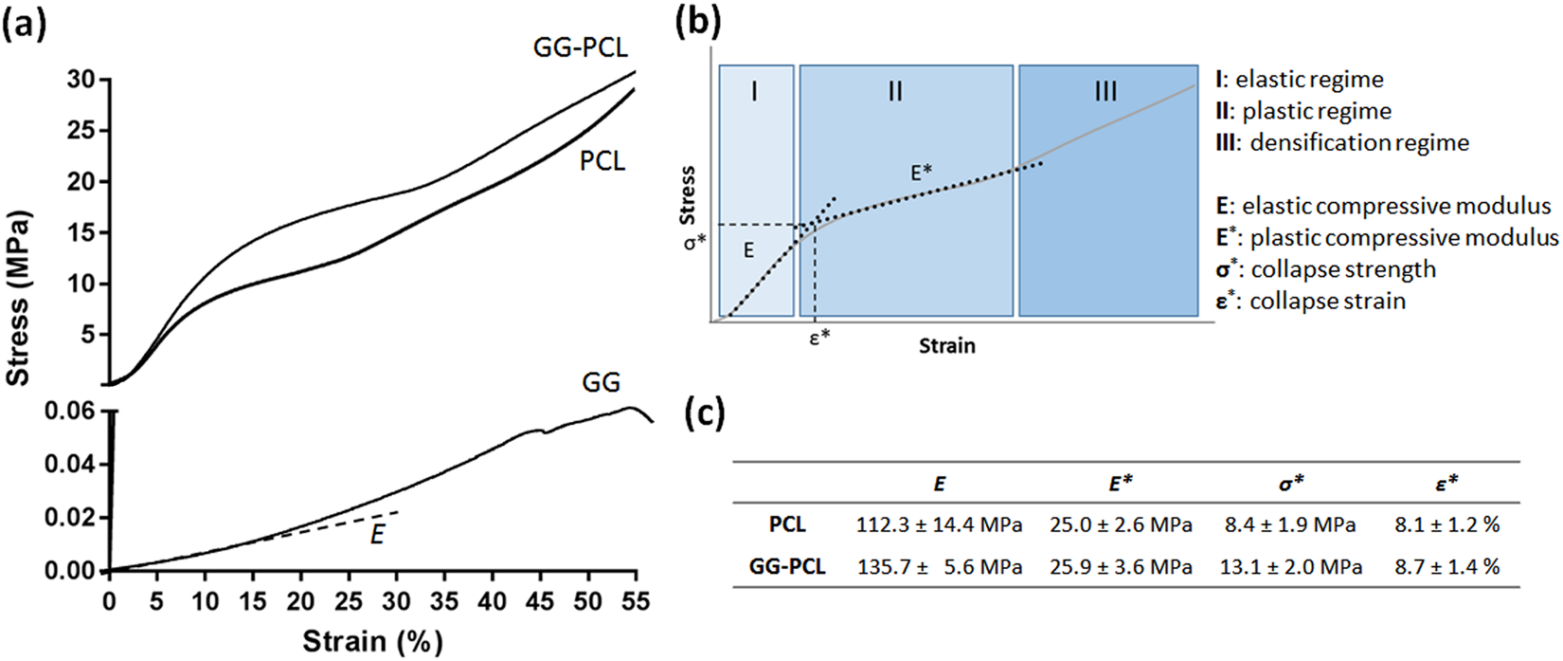
(**a**) Stress-strain curve obtained by static compression test. (**b**) Schematic representation of σ/ε profile for the evaluation of elastic compressive modulus (E), plastic compressive modulus (E*), collapse strength (σ*), collapse strain (ε*). (**c**) Calculated values for PCL scaffolds and GG-PCL constructs.

Figure 4(a) shows the stress-strain curve obtained by the compression test at strain of 0–55%.

**Figure 5:**
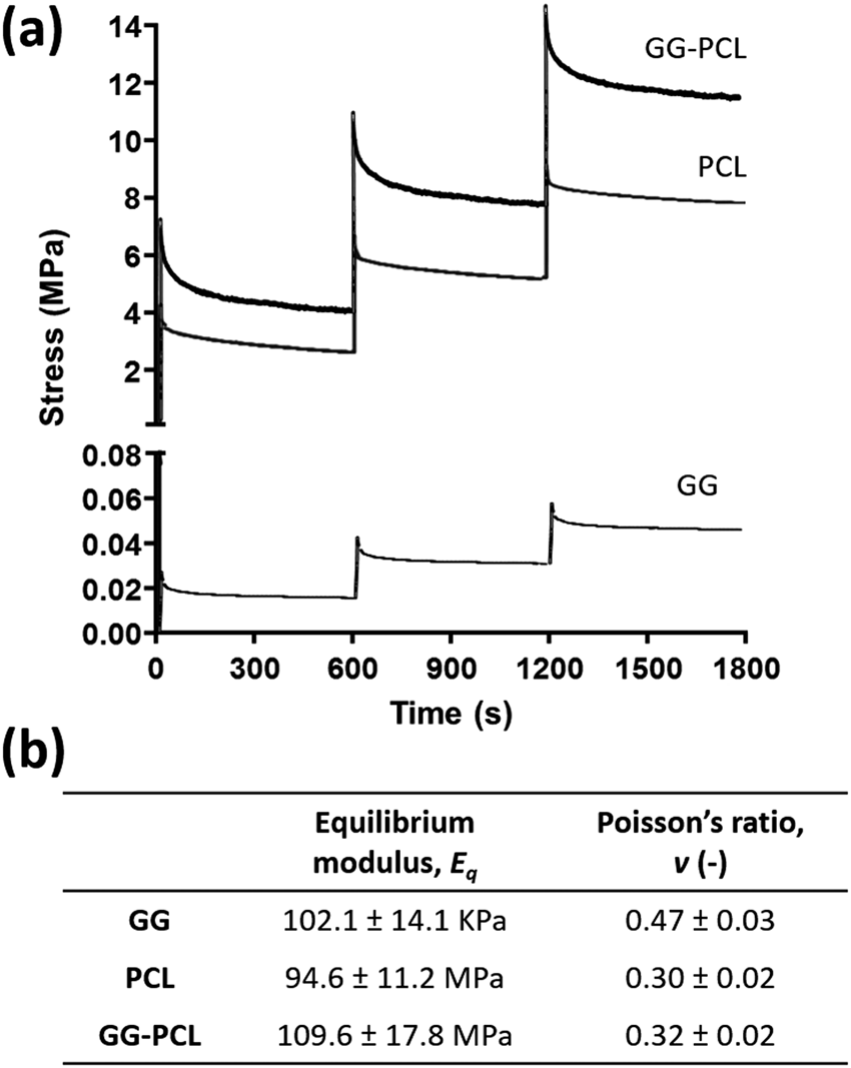
(**a**) Typical stress-behaviour of GG hydrogel, PCL scaffolds and GG-PCL constructs in response to ramp displacements. (**b**) Unconfined equilibrium moduli (*E*_*q*_) and Poisson’s ratio (*v*) of GG, PCL and GG-PCL samples.

PCL scaffolds and GG-PCL constructs exhibited a very flat and low stress profile (called “toe” region) at a compressive strain in the range 0–1.5%, followed by a substantial increase with increasing the compressive strain (ranging from 1.5 to 10%, called “heel” region), where the materials showed a linear elastic behaviour, a collapse region (from 10 to 25–30%) where the plastic behaviour could be observed, and, finally, a densification regime where the macro- and micro- structures were compromised by the compression (Fig. 5(b)). Compressive modulus (E), collapse strength and strain (σ^∗^ and ε^∗^, respectively) and collapse modulus (E^*^) were measured from the stress–strain curve. GG-based hydrogels showed a similar trend but without an evident transition from the elastic to plastic regime (around 12–15% strain). Compressive modulus of hydrogels was 125.5 ± 5.8 kPa that is higher than the values reported in literature for tissue regeneration for other hydrogels^39,40^. The addition of Sr^2+^ for the ionic-crosslinking determined a higher compressive Young’s modulus 1.5 fold than the value reported by the same authors in a previous work, where they described a composite hydrogel, based on gellan gum and HNT as filler, but ionically crosslinked with the diffusion of Ca^2+^ in the polymeric matrix^25^. Then, compressive moduli of PCL scaffolds and GG-PCL constructs were 112.3 ± 14.4 MPa and 135.7 ± 5.6 MPa respectively. Remarkably, the incorporation of the gellan gum gel into the construct increased 1.3 fold the compressive modulus of the composite construct with a shift in the point of transition from linear to the collapse regime to higher values (from 8.4 ± 1.9 MPa to 13.1 ± 2.0 MPa for σ^∗^ and from 8.1 ± 1.2% to 8.7 ± 1.4% for ε*). The obtained values are higher or within the range of mechanical properties described in literature. As example, 3D porous PCL scaffolds proposed by Albrecht *et al*.^20^ and obtained using the MakerBot Replicator 2X showed a lower compressive Young’s modulus (44.1 MPa) due to the higher volumetric porosity of the initial design. Moreover, natural bone is a composite typically consisting of cortical and cancellous bone characterised by a compressive modulus of 130–220 MPa and 5–10 MPa, respectively^41^. As reported here, the compressive results for the GG-PCL construct had moduli falling well within the target zone required for bone tissue engineering.

To compare the time-dependent mechanical properties of the proposed scaffolds, step-wise stress-relaxation experiments were performed, consisting of 3 steps of 4% compressive strain ramps, each followed by an equilibrium period in an unconfined compression. Figure 5(a) shows the stress vs. time (σ/t) profiles. All the constructs did not show a high degree of relaxation over time, however GG-PCL construct showed a sharper stress decay after each compression ramp and it reached the equilibrium with rapid relaxation than PCL alone. It is reported in literature that high degree of stress relaxation upon loading may enhance cell behaviour in terms of spreading, proliferation and differentiation of mesenchymal stem cells^42^. The resulting E_q_ of PCL scaffolds and GG-PCL constructs were lower than the values for natural bone (ranging from 230 to 900 MPa)^43^. The equilibrium modulus is an index for understanding the viscoelasticity of a biomaterial, fundamental for the design and fabrication of a scaffold^44^.

Finally, Poisson’s ratio, a material property which is defined as the negative ratio of transverse to longitudinal strain, of the scaffolds was measured during the stepwise stress-relaxation tests. Figure 5(b) shows that all the constructs had a good volumetric conservation in long-term loading, in particular for the GG hydrogels *v* was close to 0.5 (0.47 ± 0.03). The measured Poisson’s ratio values were added as input for the following computational modelling.

### Finite Element Method (FEM) analysis

**Figure 6:**
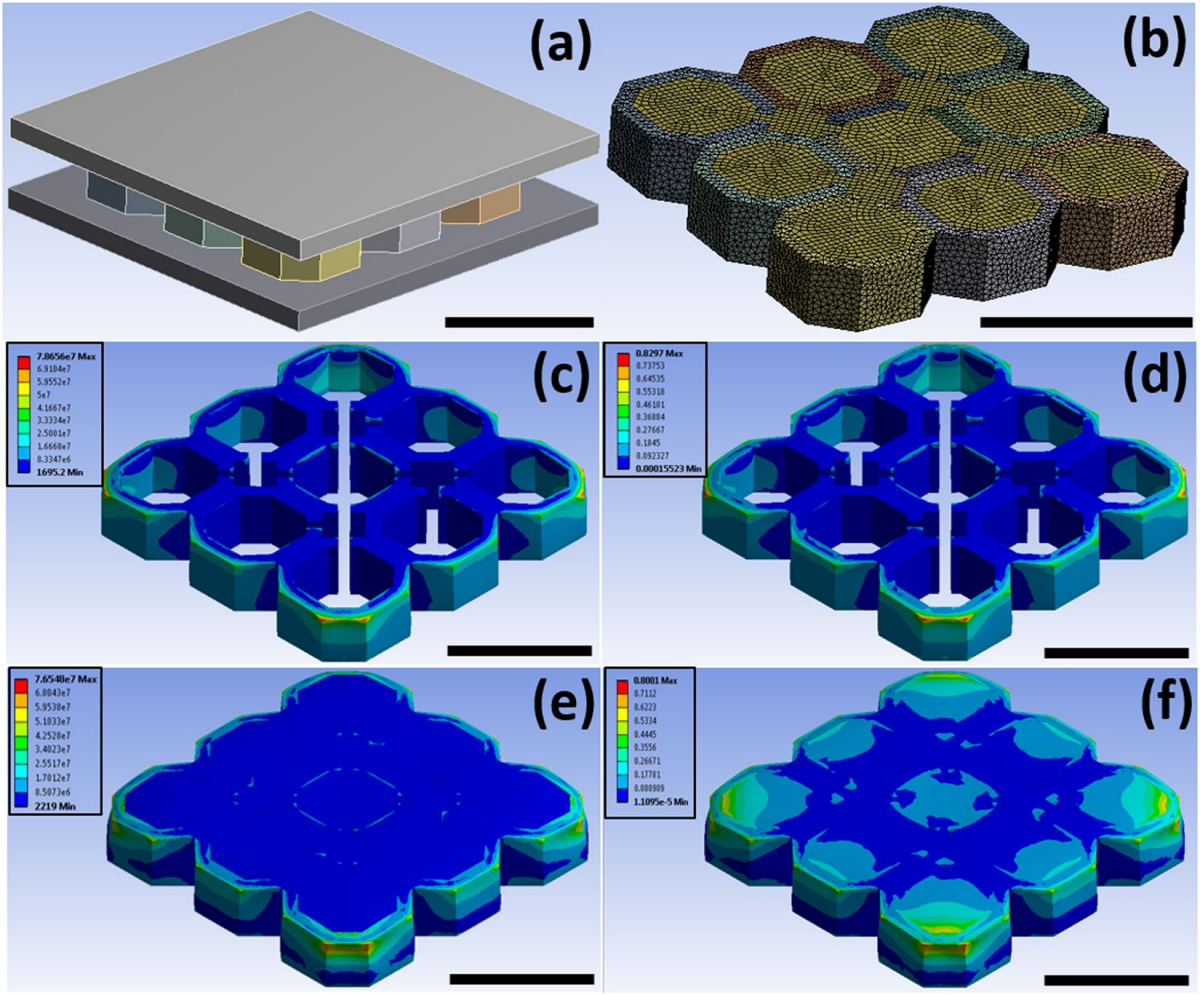
Finite element method (FEM) analysis. (**a**) Schematic representation of the simulation with two loading blocks used to compress the scaffold model; (**b**) The resulting mesh of the two geometries to be considered; (**c**) FEM-generated Von Mises stress contours for PCL scaffold; (**d**) FEM-generated total strain contours for PCL scaffold; (**e**) FEM-generated Von Mises stress contours for GG-PCL construct; (**f**) FEM-generated total strain contours for GG-PCL construct. Scale bars = 1 mm.

To further advance the insights into the reinforcement mechanism of the gellan gum-based hydrogel, a numerical model was developed using finite element method simulation. Each sample (with or without GG reinforcement) was placed between two parallel rigid plates (Fig. 6(a)), already in a frictionless contact with the structure from the beginning. One of the rigid plates was fixed while the other moved under the action of an applied force. The evolution of Von Mises stresses and total strains were recorded during the simulation (See also Supplementary Data Movies 1–6). Figure 6(c–f) shows the stress contours generated when a compressive load of 500 N was applied. The FEM simulation indicates that the scaffold underwent a higher maximum stress when without the gel (78.7 MPa respect with 76.5 MPa for the GG-PCL), with a higher stress distribution along all the scaffold height. This phenomenon demonstrates the high tendency of the GG hydrogels as reinforcement with far superior strength properties under compressive load. Furthermore, due to the idealization of the geometry and the absence of plasticity in the simulations, the GG-PCL construct showed a higher stiffness *in silico* compared to experiments (140 MPa respect with 109 MPa). The obtained values of compressive modulus are both similar to those available in tibia and vertebrae according to the data reported by Lakatos^45^. Regarding the strain contours, GG-PCL construct exhibited higher maximum strain value (0.80 m/m respect with 0.83 m/m for the PCL alone) showing a higher tendency of elastic deformation of the reinforced-construct. The surface strains calculated are the ones that should be felt by the cells attached to the scaffold, as suggested by previous simulations of mechano-regulation studies^46,47^. Therefore, all this FEM insights may imply as interesting application in Bone Tissue Engineering for the development of bioreactors, that are mechanical devices able to regulate mechanical/electrical stimuli applied on cells/scaffolds for a particular application in tissue engineering^48^.

### Cytocompatibility assessment

**Figure 7:**
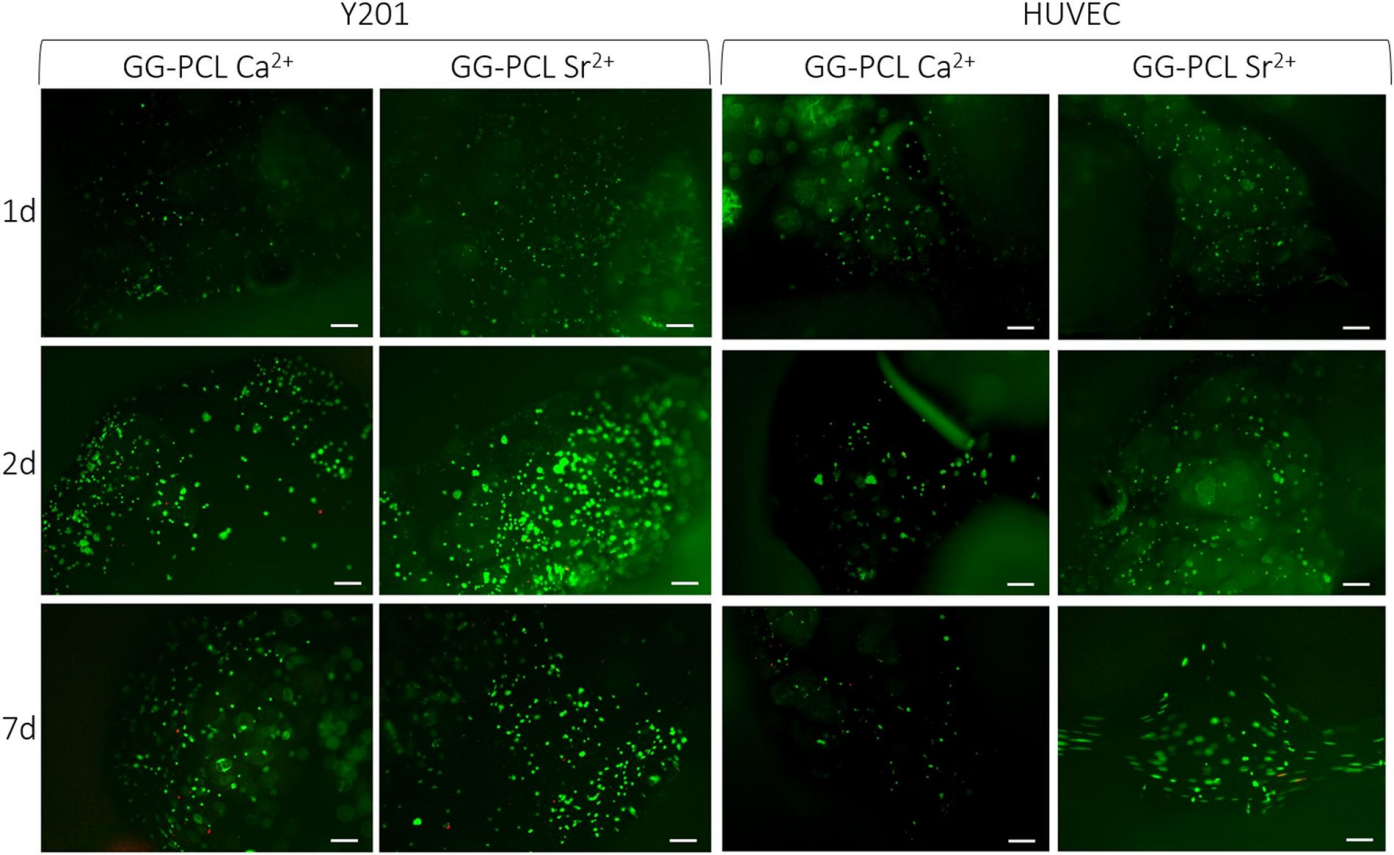
Live/Dead assay performed on the GG-PCL constructs crosslinked with Sr^2+^ or Ca^2+^ ions after 1, 2 and 7 days of culture of Y201 and HUVEC cells. Scale bars: 10 μm.

**Figure 8:**
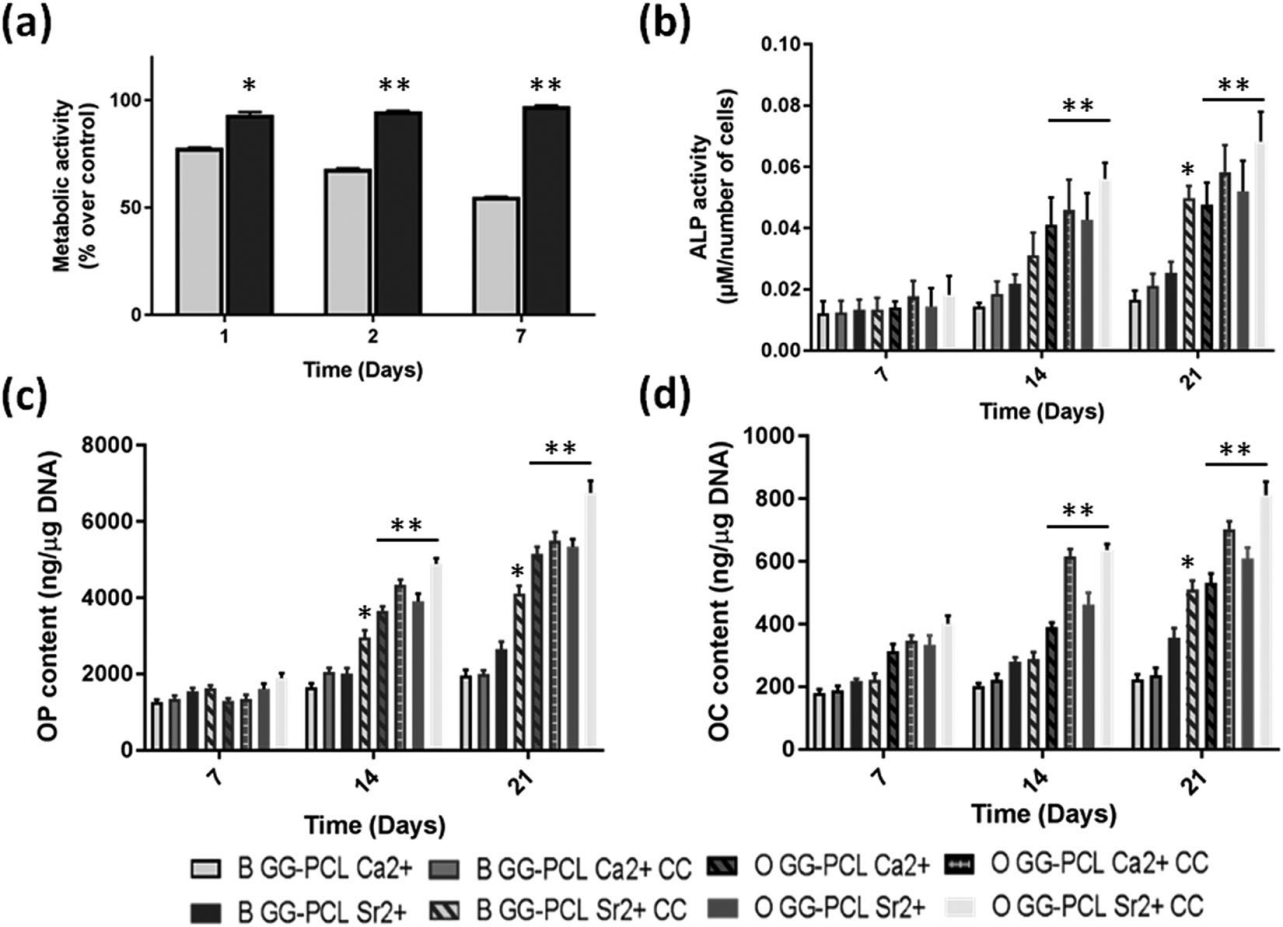
*In vitro* cell tests. (**a**) Cell metabolic activity (PrestoBlue assay) after 1, 2 and 7 days within GG-PCL scaffold crosslinked with Sr^2+^ or Ca^2+^ ions. (**b**) Intracellular alkaline phosphatase activity of Y201s after culturing either with basal (coded as B) or osteogenic media (coded as O) for 7, 14 and 21 days without or with co-culture with HUVEC (coded as CC). (**c**) Osteopontin protein content of Y201 cultured either with basal (coded as B) or osteogenic media (coded as O) for 7, 14 and 21 days without or with co-culture with HUVEC (coded as CC). (**d**) Osteocalcin protein content of Y201 cultured either with basal (coded as B) or osteogenic media (coded as O) for 7, 14 and 21 days without or with co-culture with HUVEC (coded as CC). For (**b**,**c**,**d**) the statistic significance is in respect to each composition control in basal media without HUVEC for each time point (*p < 0.05 and **p < 0.001).

Human TERT immortalised bone marrow stromal cell line (Y201) and human umbilical vein endothelial cells (HUVEC) were encapsulated within the hydrogels and embedded in the 3D printed PCL scaffolds. The overall cell viability and metabolic activity was evaluated, comparing the effect of Sr^2+^ ions crosslinking with that of Ca^2+^ ions (Figs 7 and 8). The Live/Dead assay performed at days 1, 2 and 7 demonstrated a uniform viable cells distribution of both encapsulated cytotypes at each time point tested (Fig. 7). The constructs crosslinked either with Sr^2+^ or Ca^2+^ ions displayed similar Y201 viability by Live/dead assay. However, variances on encapsulated HUVEC cells viability is observed between Sr^2+^ or Ca^2+^ crosslinked hydrogel after 7days of incubation by obtaining a higher HUVEC viability in Sr^2+^ crosslinked construct. This evidence is supported by the recent work of Zhang *et al*.^49^, in which the inductive role of Sr^2+^ ions on endothelial cells proliferation and migration was described.

In Fig. 8a, the impact of Sr^2+^ ions on the overall cellular metabolic activity is reported. The PrestoBlue assay detected a higher cellular metabolic activity on Sr^2+^ crosslinked constructs, following similar behaviour observed on the two-dimensional control co-culture. Therefore, proposed construct demonstrated suitable properties as potential 3D *in vitro* co-culture system without negatively affecting cellular response within a week of incubation.

Moreover, the change of the crosslinker ion had a clear impact on cellular metabolic activity. Indeed, cells encapsulated within the constructs crosslinked with Ca^2+^ ions underwent a progressive decrease of metabolic activity over time, from 78% over control at day 1 to 68% at day 2 and 55% at day 7. These findings could be partially explained with the effect of tested crosslinker ions on innate biochemical activity of cells. However, since the constructs crosslinked with Sr^2+^ and Ca^2+^ showed different mechanical performances, an indirect effect of the hydrogel stiffness on cell behaviour could also be relevant^50–52^.

In order to evaluate the differentiation of the Human TERT immortalised bone marrow stromal cells encapsulated in the GG-PCL models, ALP activity, OPN and OCN expression were evaluated. As control, for testing the effect of the endothelial cells on the Y201 behaviour, GG-PCL samples incorporated with Y201 only (thus without HUVEC) were considered as control. It is commonly accepted that ALP changes are related with functional activity of the bone-derived cells, such as their osteogenic differentiation and the mineralisation onset^53,54^. For executing a preliminary evaluation on the capacity of the proposed model to induce osteogenic differentiation, we cultured Y201 into the GG-based gels and quantified the ALP activity after 7, 14 and 21 days according to different culture conditions: in basal or osteogenic media, with or without (as control) the presence of HUVEC cells. Not surprisingly, as shown in Fig. 8b, the ALP activity showed significantly higher levels when cells were cultured under osteogenic media rather than basal media. However, at day 14 and 21 ALP activity on the samples cross-linked with Sr^2+^ under basal media was significantly higher than the sample cross-linked with Ca^2+^. It has been demonstrated and reported in literature that the release of chemical species containing Silicon (Si), Boron (B) or Sr could stimulate the osteoblast proliferation and, particularly their differentiation^55^. As example, Ren *et al*. showed that PCL electrospun membranes incorporating Sr-substituted bioactive glass particles enhanced the ALP activity of MC3T3-E1 cells in the presence of osteogenic media in comparison with the glass-free PCL scaffolds after 21 days of culture^56^. However, as recently described by Aimati *et al*. lower concentrations of strontium facilitate osteogenic differentiation of human adipose-derived stem cells (hASCs) (<250–500 µM), while higher doses cause hADSCs apoptosis with activation of the ERK1/2 signalling pathway contributing to this process^57^. Furthermore, it was observed higher ALP activity in Y201/HUVEC co-culture in comparison with Y201 cells that is related to a positive effect of endothelial cells. Our results are in agreement with that reported in 2018 by Chen *et al*. that have investigated the effects of co-culture of endothelial cells (ECs) with different types of MSCs on bone regeneration in calcium phosphate cement scaffolds^58^. In their study, they described that, under co-culturing conditions, all types of MSCs on the scaffolds successfully went into the osteogenic differentiation pathway, with high expressions of osteogenic markers (ALP, OCN, and Collagen I). The ECs in all the co-cultured constructs underwent angiogenic differentiation, with high expressions of vascular endothelial cadherin, which is indispensable for proper vascular morphogenesis and serves the purpose of maintaining the newly formed vessels^59^.

Although the samples containing Ca^2+^-crosslinked hydrogel were not capable of inducing the ALP protein expression alone, they were capable of improving the ALP expression during the differentiation process of Y201 to osteoblasts under osteogenic media condition for 21 days of culture.

In addition to the reported biological data, the differentiation level of Y201 under basal or osteogenic media was evaluated by quantitative expression of two major bone-specific proteins, osteopontin (OPN) and osteocalcin (OCN). The relative expression of those proteins was normalised in respect with the cell proliferation (Fig. 8c,d). It is well reported in literature that OPN, synthesised by bone forming cells, is responsible for cell attachment, proliferation, and, mainly, Extracellular mineralisation^60^; while OCN, a bone-specific glycoprotein capable of binding with calcium, is able to promote ECM calcification^61^. As reported for ALP activity, OPN and OCN evaluation showed significantly higher protein expression levels when Y201 were cultured under osteogenic media rather than basal media. In the case of OPN, at day 7 there was no significant difference in OPN expression in all samples under basal and osteogenic conditions, however at day 14 it was detected a highest expression peak for osteogenic media cultures, indicating the start of the mineralisation phase. To emphasise, Sr^2+^-crosslinked GG-PCL samples under basal condition showed a significant overexpression of OPN protein at day 21 (*p < 0.05 for Y201 culture and **p < 0.001 for Y201/HUVEC co-culture respectively), supporting the higher ALP activity and the formation of mineral deposits stained by Alizarin Red (Fig. S1).

For OCN evaluation, Fig. 8d shows a high protein expression up to day 21, with a similar trend discussed for OPN expression. Differently, we noticed only a significant difference of OCN expression in GG-PCL Sr^2^ samples under osteogenic and co-culture conditions, that may indicate an early tendency to bone ECM maturation^62^. Notable is that GG-PCL Sr^2^ samples under basal media condition with or without presence of HUVEC exhibited a peak of expression at day 21, suggesting that this model was able to induce in long term OCN protein expression, which corroborates the ALP data.

## Methods

### Materials

GG (Phytagel, formula weight 1,000 kg/mol; low acylation degree), HNT, free flowing calcium chloride- Redi-dri), strontium chloride powder (≥99.99% trace metals basis), PCL pellets (2–3 mm diameter, 70–90 kDa) were all supplied by Sigma-Aldrich (Italy). The ultrapure water employed throughout the experiments was obtained with a Milli-Q Integral system equipped with a BioPak ultrafiltration cartridge (Millipore, Merck).

### PCL spool development and three-dimensional printing by fused deposition modelling

Spools were made using a Rondon 10 mm double-screw extruder. PCL pellets were added into the extruder’s hopper at 20 rpm and the shear force of the machine’s extrusion screw fed the raw material into the five heating zones. A cooler zone was located before the die, thereby causing a build-up of pressure that forced the melted polymer out of the head at around 44 rpm. The temperature of the five heating zones was kept in the range between 75 °C −120 °C. All the extruded filaments presented a diameter of 1.75 ± 0.1 mm, measured by the use of a Vernier caliper.

Before FFF process, the scaffold geometry was firstly designed by computer aided design (CAD) using Autodesk Inventor Professional 2015 software.

Spools were loaded into the MakerBot Replicator 2X (MakerBot Industries) and Symplify3D software was used to convert the. STL file generated by Autodesk Inventor in the g-code for printing. The scaffolds were printed after optimization of the following process parameters: heated build plate 40 °C, extruder temperature 140 °C, speed 25 mm∙min^−1^ and layer height 0.25 mm.

### Gellan gum incorporation within the scaffolds

The hydrogel preparation was extensively described in a previous work^25^. Briefly, GG powder (2% w/v) was added to an aqueous solution under stirring at 90 °C. A water suspension of HNT (0.5% w/v) was sonicated for 15 min and mixed with gellan to obtain the hydrogel. The latter was rapidly pipetted within the PCL scaffold, by adding 20 μL into the big pores and 10 μL into the small ones) to obtain the GG-PCL construct. To achieve the hydrogel crosslinking with Sr^2+^ ions, an external gelation method with SrCl_2_ impregnated membranes was exploited^63^. An aqueous solution of SrCl_2_ 25 mM was used as source of Sr^2+^ ions. As control, a CaCl_2_ solution (2.5 mM) was utilised to induce GG gelation.

### Morphological characterisation

Stereomicroscopy (Leica DM IL LED) in bright field mode was used for investigating the final morphology of the PCL scaffold and GG-PCL construct at 1.5X magnification. The pore size was calculated by ImageJ software.

### X-ray photoelectron spectroscopy

XPS analysis of GG-PCL constructs was carried out using a scanning microprobe PHI 500 VersaProbe II (Physical Elecronics, Chanhassen, MN), equipped with a monochromatised AlKα X-ray radiation source. The samples were analysed in HP mode (scanned size about 1400 × 200 µm). Survey scans (binding energy (BE) range 0–1200 eV, FAT mode, and pass energy 117.4 eV) and high-resolution spectra (FAT mode, pass energy 29.35 eV) were recorded. Data analysis of the latter was performed using a non-linear least-square fitting program, i.e. MultiPak software package (version 9.6.1.7). The experimental points of the detailed spectra were fitted using Gaussian-Lorentzian peaks having the same full width at half maximum (FWHM). Charge referencing was performed by setting at 284.8 eV the lower binding energy C1s photo-peak (i.e. C1s hydrocarbon peak). Quantification of each element (reported as atomic percentage, At%) was made using normalised peak area. The normalization of the peak area and comparison of data from different elements was enabled by correction with empirically derived sensitivity factors according to MultiPak library.

### Water uptake evaluation

Dried polymeric samples were firstly weighed (m_di_), successively placed in previously weighed (m_b_^t^) tea bags. Tea bags containing the samples were then sealed and soaked in PBS to determine the water uptake profile up to 24 h at 37 °C. Therefore, samples in tea bags were weighed prior (m_i_^0^) and after predetermined time intervals (m_i_^t^). Water excess was removed by placing the tea bags on a drying rack for 1 minute. For each sample, the water uptake percentage (Wu%) along the time was calculated using Equation (1) reported below:

1 $${({\rm{Wu}} \% )}_{{\rm{i}}}^{{\rm{t}}}=[({{\rm{m}}}_{{\rm{i}}}^{{\rm{t}}}-{{\rm{m}}}_{{\rm{b}}}^{{\rm{t}}})-{{\rm{m}}}_{{\rm{i}}}^{0}]/{{\rm{m}}}_{{\rm{di}}}\times 100$$

The measurements were performed in triplicate and results were reported as mean ± standard deviation.

### Mechanical testing and analysis

Mechanical tests of the PCL scaffolds and GG-PCL constructs were performed using the EZ-SX mechanical testing equipment (Shimadzu, Japan). Five samples for each type were used to evaluate the compression resistance at room temperature. The crosshead speed was set at 0.015 mm∙s^−1^ with a tare load of 0.5 N. The compression plates have been equipped with microporous fritted glass which would allow the hydrogel moving out under compression in order to avoid secondary hydrostatic pressure. The compressive modulus was calculated as the slope of the initial linear portion of the stress–strain curve (0–10%), while the values of plastic compressive modulus, collapse strength and strain (E*, σ* and ε*, respectively) were measured as described by Gentile *et al*.^64^. As control, GG hydrogels (diameter of 1.6 cm and height of 2 cm) were also tested with a preconditioning of 0.05 N using the same process parameters described before.

Furthermore, stress-relaxation tests were performed to evaluate the equilibrium behaviour of the samples as described by Bas and Hutmacher^28^. All the constructs were subjected to 4% ramp compressive strain steps, each followed by a relaxation period of 10 minutes up to reach 12% strain. The equilibrium modulus (*E*_*q*_) was determined as slope of the stress-strain curve. The lateral changes of the construct (observed at the end of each tests compared with the initial conditions) were used to calculate the Poisson’s ratio (*v*) in equilibrium.

### FEM analysis

The numerical experiments for validating the proposed *in vitro* model were conducted using the FEM. Particularly, in order to analyse the compression behaviour of the scaffold and gel, two loading blocks or plates were used to compress the scaffold model. The contact between the plates was considered as frictionless. While the bottom plate is fixed, the top plate was allowed to move vertically under the applied force. A force up to 500 N was used throughout the simulations. The height of each plate was set as 40% of the total scaffold height to ensure uniform stress distribution around the scaffold-loading block region. Thus, the scaffold height was set at 2 mm, while the plate height at 0.8 mm. The two solid models, mimicking the 3D printed PCL scaffold and the GG based hydrogels, were assumed to be isotropic, with a linear elastic behaviour.

Mathematical models will be devised for the geometry described above. 3D printed PCL scaffold was meshed with 0.2 mm tetrahedron elements, while the hydrogel was meshed uniformly with hexahedral elements with size of 0.2 mm assuming that an uniform cells distribution into the hydrogel with an initial constant contraction across the entire volume^65^. This has resulted in a total of 90107 elements. The static structural analysis system of the finite element solver, ANSYS Mechanical APDL software, was used to produce the meshes described above and for all the following FEM analysis. Finally, specific material and mechanical parameters were adopted in this study. Experimental data were used as the input for the simulations: the elastic modulus and yield strength of the PCL scaffold was set as 112 MPa and 9 MPa respectively based on the best result obtained through mechanical compression tests, while a density of 1.145 g/cm³ and a Poisson’s ratio of 0.3 (calculated in the stress relaxation tests) were also considered. For the hydrogel, the compressive modulus of the hydrogel was 126 kPa with a yield strength of 58 kPa. The density was set to 0.98 g/cm³ due to the fact that the high content of water within the gel with a Poisson’s ratio of 0.47, while considering that the hydrogels behaves as an almost incompressible material when loaded at high strain rates.

### Cell culture

Human TERT immortalised bone marrow stromal cell line (Y201) were kindly supplied by Prof P. Genever (York University) and cultured as already described^66^. Briefly, cells were grown at 37 °C, 5% CO_2_, in Dulbecco’s Modified Eagle Medium (DMEM, Sigma) with low glucose content, supplemented with 15% fetal bovine serum, 2mM L-glutamine and a 1% penicillin-streptomycin mixture (100 U/mL). Human umbilical vein endothelial cells (HUVEC) were cultured as recommended by the seller (Lonza Biosciences, Switzerland). Cells were kept at 37 °C, 5% CO_2_ and cultured in Medium 200 without phenol red, supplemented with Low Serum Growth Supplement (Gibco).

### Cell encapsulation into hydrogels

The hydrogel solution was stirred and slowly cooled until a temperature of 37 °C was reached. Each cell type was re-suspended in its medium (DMEM for Y201, Medium 200 for HUVEC), mixed 1:1 with the hydrogel solution, and drop casted in the PCL-grid as described in Fig. 1. The cell density was 2 × 10^5^ cells/mL. When the gelation occurred, the encapsulated hydrogels were crosslinked with SrCl_2_ through the external gelation method^63^. Then, the whole construct was covered with additional medium, i.e. DMEM:Medium 200 in a mixture 1:1 and incubated at 37 °C.

### Biological assessments: metabolic activity and viability of encapsulated cells

The live/dead staining (Live/Dead Cell Staining Kit II, PromoKine, PromoCell GmbH, Germany) was exploited to study cell viability at 1, 2 and 7 days. The GG-PCL constructs were washed with PBS and stained for 40 min with a solution of Calcein-AM and Ethidium homodimer III in PBS prepared according to the seller. Samples were imaged with a Zeiss Axio Imager fluorescence microscope.

As far as the metabolic activity is concerned, the Presto Blue assay was adopted to test the metabolic activity of encapsulated cells after 1, 2 and 7 days of culture. A 10% aliquot of PrestoBlue was added to the samples and incubated for 40 min at 37 °C. The fluorescence was measured by a Varian Cary Eclipse Fluorescence spectrophotometer (Perkin Elmer, Waltham, MA), setting the excitation and emission wavelength at 560 and 590 nm, respectively. The results were corrected for fluorescence of control wells and expressed as percentage of the positive control co-culture, consisting of Y201 and HUVEC cells seeded on Tissue Culture Plates (TCPs).

After 21 days of cell culture Y201 differentiation was evaluated in basal (as described before) and osteogenic medium (after 4 days of cell seeding consisted of basal medium plus 50 μg/mL ascorbic acid, 10–8 M dexamethasone (Sigma) and 10 mM β-glycerophosphate (Fluka Biochemika)). Alkaline Phosphatase activity (ALP) was evaluated after 7, 14 and 21 days by adding 500 µL alkaline buffer solution and 0.5 mL of stock substrate solution (40 mg p-nitrophenyl phosphate disodium, Sigma) to 100 µL of each lysate samples (obtained following the same protocol described below for the PicoGreen assay), diluted in 10 mL of distilled H_2_O for 1 h at 37 °C. The p-nitrophenol production was analysed by monitoring the solution absorbance using Leica DM2500 at 410 nm. PicoGreen dsDNA reagent (Invitrogen) was used to calculate the cell number for each sample in order to make a correct normalisation of the ALP absorbance values. In essence, after culturing period, the samples were washed with PBS and then incubated at 37 °C for 3 h followed by several freezing at −80 °C /thawing steps in ultra-pure water (1 mL) to ensure cell lysis. Then, 100 μl DNA samples were incubated with 100 μl diluted (1:200) PicoGreen reagent in 1× TE buffer in a 96-well opaque, flat-bottomed assay plate. The assay was performed according to the manufacturer’s protocol. The fluorescence was read at excitation 485 nm and emission 525 nm and was compared with a DNA standard curve provided with the kit. The mean ± standard deviation were calculated for five tests.

Osteopontin (OPN) and osteocalcin (OCN) protein expression of Y201 was assessed by immunoassay technique to evaluate the osteoblast differentiation, following the protocol reported by Fernandes *et al*.^67^. The concentration of OPN and OCN was determined for all time culture periods, using the lysates used for DNA quantification by Picogreen. OPN quantitative determination was performed by the use of Mouse/Rat Osteopontin Quantikine ELISA Kit (R&D Systems, UK). In brief, 50 µL of assay diluent RD1W and 50 µL of standard (2500 to 39 pg/mL), control and sample were added into to the multi-well plate and kept to incubate at 25 °C for 2 h. After 4 washing steps, 100 µL of Mouse/Rat OPN conjugated were added and incubated at 25 °C for 2 h. The sandwich complex was rinsed 4 times in order to react with 100 µL of substrate solution before adding 100 µL of stop solution. Finally, the optical density was determined at 450 nm and concentration of OPN obtained from standard curve plot. OCN quantitative determination was performed by the use of Rat Bla-Osteocalcin High Sensitive EIA kit (Takara Clontech, Japan). In brief, 100 µL of samples and standard solution (16 to 0.25 ng/mL) were incubated for 1 h at 37 °C with the capture-antibody, rat osteocalcin C-terminus-specific antibody. After OCN capture and 3 washing steps, 100 µL of the enzyme-labelled antibody (GlaOC4–30) specific to Gla-OCN was incubated at room temperature for 1 h. The sandwich complex was rinsed 4 times and allowed to react with 100 µL of substrate solution for 10–15 min. Finally, after adding the stop solution the optical density was determined at 450 nm and concentration of OCN obtained from standard curve plot. OPN and OCN content was calculated by normalising OPN or OCN concentration per DNA concentration for each condition and time point.

Mineral deposits was determined by Alizarin Red staining. Wash water was carefully aspirated from wells and samples were incubated with 300 μl of 2% (w/v) Alizarin Red solution (pH 4.2) at room temperature for 30–45 min. Alizarin Red Solution was then removed and samples were washed several times with distilled water. Calcium deposits were stained orange/red by Alizarin Red solution and visualised by bright field microscopy (DMLB-Leica Fluorescent light Microscope combined with Camera-Advanced SPOT).

One-way ANOVA with repeated measurements was performed with GraphPad Prism 7.01 software to assess the statistical significance of the PrestoBlue results. The Bonferroni’s test was exploited to compare the impact of the different variability sources. Statistical significance was declared at *p < 0.05 and **p < 0.001.

## Conclusions

To our knowledge, this work is the first to present the combination of FFF method and hydrogel encapsulation that allows the fabrication of an intricate and attractive construct in order to meet the mechanical and physico-chemical requirements of an *in vitro* bone model. The obtained multi-compartmentalised construct was composed of a 3D-printed PCL porous scaffold filled with a GG-based hydrogel. The GG-PCL construct exhibited enhanced physical performances and the computational simulation was in line with the experimental findings. Results evidenced a high cell viability and metabolic activity of both, human-TERT mesenchymal stem cells (TERT- hMSCs) and human umbilical vein endothelial cells (HUVECs), when encapsulated within GG hydrogel, disclosing the positive and robust effect of Sr^2+^ ions used as ionic GG-crosslinker. Moreover, fabricated inter-connected compartmentalised scaffolds containing encapsulated TERT- hMSCs and HUVECs into GG-hydrogels showed a greater degree of osteogenic differentiation in the Sr^2+-^crosslinked GG-PCL scaffold compared to Ca^2+^-crosslinked control gels. The established procedure provides an innovative avenue for *in vitro* engineering of tissue constructs with complex structure and function, with the great potential to be used as *in vitro* experimental model for future *in vivo* therapeutic bone replacements and drug testing, such as for alveolar bone that is extremely affected during periodontal disease.

## Electronic supplementary material

Supplementary information

Movie 1

Movie 2

Movie 3

Movie 4

Movie 5

Movie 6

## Data Availability

The datasets generated and/or analysed during the current study are available from the corresponding authors on reasonable request. See Supplementary Information for the movies acquired within FEM analysis.
