# Supplementary material for: Multi-compartment scaffold fabricated via 3D-printing as in vitro co-culture osteogenic model
Source: Sci Rep. 2018 Oct 11;8:15130. doi: 10.1038/s41598-018-33472-1 (PMC6181937; doi:10.1038/s41598-018-33472-1)
Supplement: Supplementary file 1 — Supplementary information [file 41598_2018_33472_MOESM1_ESM.pdf]

Multi-compartment scaffold fabricated via 3D-printing as *in vitro* co-culture osteogenic model

Elvira De Giglio[1*], Maria A. Bonifacio[1], Ana M. Ferreira[2], Stefania Cometa[3], Zhi Yuan Ti[2], Antonella Stanzione[1], Kenny Dalgarno[2], Piergiorgio Gentile[2*]

[1] Dept. of Chemistry, University of Bari Aldo Moro, Via E. Orabona 4, Bari, 70126, Italy

[2] School of Engineering, Newcastle University, Stephenson Building, Claremont Road, Newcastle upon Tyne, NE1 7RU, UK

[3] Jaber Innovation s.r.l., via Calcutta 8, Rome, 00144, Italy

Corresponding author e-mails: *piergiorgio.gentile@ncl.ac.uk and elvira.degiglio@uniba.it

Supplementary data

Figure S1. Mineral deposits stained by Alizarin-Red after 21 days of cell tests on GG-PCL constructs crosslinked with Sr^{2+} under basal media (**a**) without and (**b**) with co-culture with HUVEC, and under osteogenic media (**c**) without and (**d**) with co-culture with HUVEC. Scale bars correspond to 500 μm.

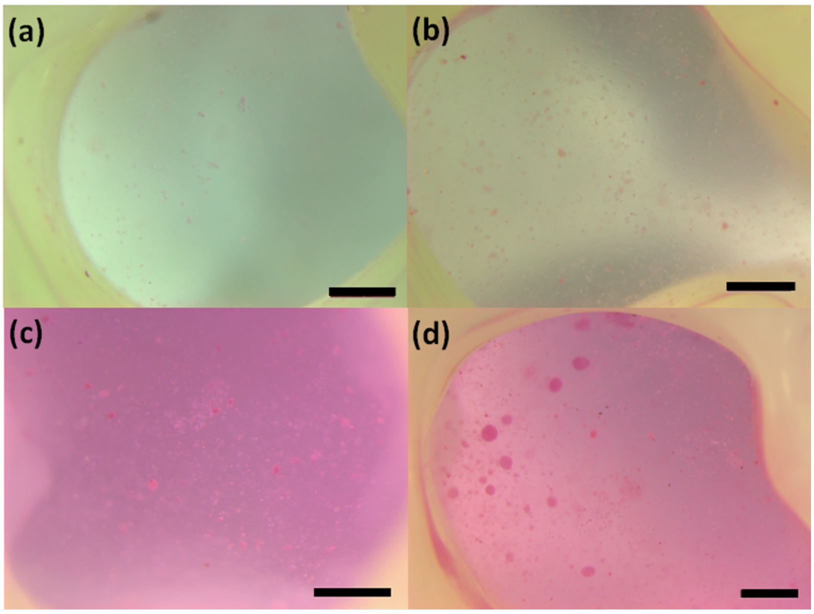

Movie 1. Equivalent von Mises stress distribution of PCL scaffold obtained by ANSYS Mechanical APDL software.

Movie 2. Equivalent total strain distribution of PCL scaffold by ANSYS Mechanical APDL software.

Movie 3. Equivalent total deformation distribution of PCL scaffold by ANSYS Mechanical APDL software.

Movie 4. Equivalent von Mises stress distribution of GG-PCL scaffold by ANSYS Mechanical APDL software.

Movie 5. Equivalent total strain distribution of GG-PCL scaffold by ANSYS Mechanical APDL software.

Movie 6. Equivalent total deformation distribution of GG-PCL scaffold by ANSYS Mechanical APDL software.
